# The Role of Intestinal Microbiota in Modulating Mesenchymal Stromal Cell Therapy in a Murine Model of Lupus-like Autoimmunity

**DOI:** 10.1007/s43657-025-00303-z

**Published:** 2026-09-07

**Authors:** Yuxuan Chen, Shuai Ding, Yang Hang, Rujie Zhu, Zhong Liu, Keren Yang, Xuefeng Gao, Lingyun Sun

**Affiliations:** 1 Department of Rheumatology and Immunology, Nanjing Drum Tower Hospital, Affiliated Hospital of Medical School, Nanjing University, Nanjing, 210008 Jiangsu China; 2 Department of Rheumatology and Immunology, Nanjing Drum Tower Hospital Clinical College of Nanjing Medical University, Nanjing, 210008 Jiangsu China; 3 Department of General Surgery, Shenzhen University General Hospital, Shenzhen University Clinical Academy, Shenzhen, 518055 Guangdong China; 4 Integrative Microecology Clinical Center, Shenzhen Clinical Research Center for Digestive Disease, Shenzhen Technology Research Center of Gut Microbiota Transplantation, The Clinical Innovation & Research Center, Department of Clinical Nutrition, Shenzhen Hospital, Southern Medical University, Shenzhen, 510086 China

**Keywords:** Systemic lupus erythematosus, Mesenchymal stromal cell, Gut microbiota, Serum metabolites

## Abstract

This study investigated the impact of gut microbiota on the efficacy of mesenchymal stromal cell (MSC) therapy in lupus-like autoimmunity. Using a co-housing approach to facilitate microbiota transfer between mice, we assessed lupus-like phenotypes, gut permeability, 16S rRNA sequencing, and metabolomics analyses. While MSC therapy effectively ameliorated lupus-like autoimmunity, its therapeutic benefits were significantly diminished when MSC-treated mice were co-housed with untreated R848-induced lupus-like mice. Co-housing altered therapeutic outcomes, impaired intestinal barrier function, and modulated immune cell subpopulations, which correlated with changes in gut microbiota composition and serum metabolite profiles. Two key bacterial taxa (*Bacteroides acidifaciens* and a taxon from the *Muribaculaceae*) and three serum metabolites (cinnamic acid, 3,4-dihydroxyhydrocinnamic acid, and pyruvic acid) were identified as correlating with T and B lymphocyte development. In vitro experiments revealed that these metabolites suppressed T cell activation, restored the Th1/Th2 and Th17/Treg balance, and exhibited an additive effect with MSC in regulating T cell responses. These findings directly link the microbiota–metabolite axis to the modulation of MSC therapeutic efficacy, emphasizing the critical role of gut microbiota and its metabolic products in determining MSC therapy outcomes for lupus-like autoimmunity.

**Supplementary Information:**

The online version contains supplementary material available at https://doi.org/10.1007/s43657-025-00303-z.

## Introduction

The gut microbiota is crucial for maintaining host homeostasis and preventing disease. Research indicates that alterations in the gut microbiota can influence immune homeostasis by impacting the function and migration of immune cells (Macpherson et al. 2023; Neurath 2019). Moreover, in the context of type 1 diabetes, a reduction in microbial diversity coupled with an increase in *Bacteroides* species is implicated in the autoimmune destruction of pancreatic β-cells (Abdellatif et al. 2019). These observations underscore the crucial role of the gut microbiota in modulating immune responses and influencing the progression of autoimmune diseases.

Recent advances in metagenomics and metabolomics have provided deeper insights into the complexity and functionality of the gut microbiome. In a colitis model of inflammatory bowel disease (IBD), dysbiosis impaired the effectiveness of anti-TNF-α therapy. Restoring microbial balance with probiotics or fecal microbiota transplantation (FMT) improved treatment outcomes (Oka and Sartor 2020). Moreover, in melanoma patients undergoing cancer immunotherapy with immune checkpoint inhibitors (ICIs), higher levels of *Faecalibacterium* and *Ruminococcaceae* species were associated with better responses, while reduced diversity or an overabundance of pathogenic bacteria correlated with poorer outcomes (Gopalakrishnan et al. 2018). These findings highlight the importance of a balanced microbiome in enhancing the efficacy of immune-modulating therapies.

Over the past decade, umbilical cord-derived mesenchymal stromal cells (UC-MSCs) have shown promise in treating autoimmune and inflammatory diseases by modulating various immune cells (Cao et al. 2021; Huang et al. 2024; Zhao et al. 2019). However, the therapeutic effect of MSC therapy is limited by several factors (Wang et al. 2017). We noted that the therapeutic effect of MSC may be influenced by the host microbiome, especially as changes in microbial composition in different environments may affect MSC function. In diabetic NOD mice, clearing the gut microbiota enabled adipose-derived MSC to reverse hyperglycemia and insulitis by enhancing the colonic mucus layer (Lv et al. 2020). Therefore, further investigation of the interaction between MSC therapy and the gut microbiota will help to optimize MSC treatment strategies to improve their effectiveness in immune regulation and gut health recovery (Dutra Silva et al. 2021).

In this study, we tested the hypothesis that dysbiotic gut microbiota limit MSC therapy in a model of lupus-like autoimmunity. Using co-housing to facilitate microbiota transfer between diseased and treated mice, we explored the specific bacteria or metabolites involved and the most affected immune subsets to better understand and enhance the effects of MSC transplantation.

## Materials and Methods

### Mouse Models

Twenty-two female BALB/cJGpt mice (strain code: N000020), aged 7–8 weeks, were purchased from GemPharmatech Co., Ltd. (Nanjing, China). All mice were housed in a specific pathogen-free (SPF) facility under controlled conditions, including a temperature of 22–26 ℃, a relative humidity of 40%–60%, and a 12-hour light/dark cycle. Cages contained autoclaved corn cob bedding, which was changed twice a week. Mice were provided with free access to a standard SPF-grade irradiated chow and tap drinking water. The facility is routinely monitored and certified free of a comprehensive panel of pathogens, including but not limited to ectromelia virus (ECTV), mouse hepatitis virus (MHV), Sendai virus (SV), pneumonia virus of mice (PVM), minute virus of mice (MVM), murine norovirus (MNV), lymphocytic choriomeningitis virus (LCMV), *Helicobacter spp*., *Salmonella spp.*, and ectoparasites. To establish a lupus-like murine model, mice received Resiquimod (R848, MedChemExpress, Cat# HY-13740, PubChem CID: 159603) treatment twice weekly via the marginal ear vein for four weeks. Following disease induction, mice were randomized into MSC-treated (*n* = 11) and PBS-treated (*n* = 11) groups using an online random number generator. Both groups received three intravenous administrations of either MSCs (5 × 10^5^ cells/mouse) or PBS, once every two weeks. Subsequently, six mice from each group were co-housed in two IVC cages (three MSC-treated and three PBS-treated per cage) for microbiota transfer over three weeks. The remaining mice (five per group) were maintained in treatment-segregated IVC cages with no cross-group exposure. All mice were sacrificed at 19 weeks of age. The exclusion criteria were animal death before the experimental endpoint and lack of samples for data analysis. All experimental procedures were approved by the Animal Ethics and Welfare Committee of the Drum Tower Hospital, Nanjing University Medical School, approved (project ID: 20200722).

### Specimen Collection

After three weeks of co-housing, urine samples were collected from the MSC, PBS, co-housed MSC, and co-housed PBS groups. The mice were then euthanized with 5% isoflurane, and blood was collected for serum preparation. Spleens, sections of the ileum, and feces were also collected aseptically for further analysis.

### UC-MSCs Isolation and Characterization

The isolation, culture, and identification of human UC-MSCs were performed in accordance with previously established industry standards (Renesme et al. 2022; Viswanathan et al. 2019), and the study was approved by Nanjing Drum Tower Hospital''s Ethics Committee (Approval No. 202019701). After obtaining written informed consent from healthy donors, umbilical tissue segments were transported in DMEM/F-12 medium (Biochannel, Cat# BC-M-002) at 4℃ within two hours post-delivery. Tissues were first washed with PBS containing 200 U/mL penicillin-streptomycin (Gibco, Cat# 15140122). Following microdissection to remove vascular structures, the remaining Wharton’s jelly was minced into 0.5–1 cm^2^ pieces, which were used as explants. Primary cultures were established in DMEM/F-12 supplemented with 10% fetal bovine serum (Biochannel, Cat# BC-SE-FBS08) and 1% GlutaMAX (Gibco, Cat# 35050061), with medium replenishment every 72 h. Upon reaching 80%–90% confluence, passage-zero cells were passaged using 0.25% trypsin-EDTA (Biochannel, Cat# BC-CE-006) at a 1:3 ratio. Passage-two UC-MSCs underwent rigorous validation: flow cytometric analysis confirmed that the cells were positive for CD73, CD90, and CD105, and negative for CD11b, CD19, and HLA-DR. Validated cells were expanded to passage six for use in subsequent experiments.

### Histological Analysis

The harvested renal tissues were fixed in 4% paraformaldehyde, embedded in paraffin, sectioned to a thickness of 5 μm, and subsequently stained with hematoxylin and eosin (H&E) or periodic acid-Schiff (PAS). Observations were conducted using an Olympus microscope, and histological assessments of renal lesions were performed. The severity of glomerulonephritis was evaluated using a 0–4 scoring system. The scoring criteria were established based on previously published and well-accepted methodologies (Chen et al. 2020): 0, normal histology; 1, mild mesangial cell proliferation; 2, moderate increases in mesangial cell number and matrix, along with glomerular basement membrane (GBM) thickening; 3, significant increases in focal capillary cells, capillary occlusion, and GBM thickness and irregularity; 4, end-stage glomerulus with diffuse capillary cell proliferation, segmental necrosis, crescent formation, and hyalinoid transformation. Renal vascular injury was scored based on the extent of perivascular cell layers: 0, no cell layer around the vessel wall; 1, fewer than five cell layers; 2, 5–10 cell layers; 3, more than 10 cell layers.

### Flow Cytometry

Mouse spleen tissue was homogenized using a syringe plunger, while the femur was removed and pulverized in a mortar, followed by filtration through a 70 μm cell filter. Prior to staining, all cells underwent treatment with a red blood cell lysis buffer. Subsequently, the enriched immune cells were washed and resuspended in a staining buffer containing antibodies targeting T and B cells (Table S1). Cells for detecting intracellular and nuclear cytokines were treated with a fixation/permeabilization buffer (Invitrogen, Cat# 88-8824-00 and Cat# 00-5523-00) before further staining. The stained cells were then subjected to analysis using a BD LSRFortessa flow cytometer, and the resulting data were processed with FlowJo software (version 10.8.1). The gating strategy is detailed in Fig. S4.

### Cell Sorting and Differentiation Induction

The spleens of BALB/cJGpt mice and R848-induced mice were aseptically harvested, mechanically dissociated, and filtered through a 70 μm cell strainer. The resulting single-cell suspension underwent magnetic-activated cell sorting using a CD3^+^ T cell isolation kit (Eynos Lifescience, Cat# SN4101) and a naïve CD4^+^ T Cell isolation kit (Eynos Lifescience, Cat# SN4104). To evaluate the effects on T cell activation, sorted CD3^+^ T cells were plated at a density of 1 × 10^5^ cells/well and treated with MSCs and varying concentrations of metabolites. The metabolites used were trans-cinnamic acid (t-CA; MCE, Cat# HY-N0610), pyruvic acid (PA; MCE, Cat# HY-Y0781), and 3,4-dihydroxyhydrocinnamic acid (DHCA; MCE, Cat# HY-N2406). In parallel, sorted naïve CD4^+^ T cells (1 × 10^5^ cells/well) were employed to assess metabolite and MSC modulation of T cell subset differentiation (Th1/Th2/Th17/Treg). Both cell types were stimulated with CD3/CD28 Activator Beads (Eynos Lifescience, Cat# AM2001); naïve CD4^+^ T cells additionally received subset-specific cytokine induction: Th1 (10 ng/mL IL-2, 10 µg/mL anti-IL-4 and 10 ng/mL IL-12), Th2 (10 µg/mL anti-IFN-γ and 10 ng/mL IL-4), Th17 (10 ng/mL IL-6 and 0.5 ng/mL TGF-β1), and Treg (10 ng/mL IL-2 and 5 ng/mL TGF-β1). MSCs, pre-stimulated with 20 ng/mL IFN-γ for 24 h, were co-cultured with T cells at a ratio of 1:10 (MSCs: T cells). After 72 h, cells were analyzed by flow cytometry.

### Immunofluorescence

Ileal tissue was first washed with PBS and then fixed in 4% paraformaldehyde. Subsequently, the tissue was permeabilized with 0.1% Triton X-100 and incubated with an anti-Occludin primary antibody (Abcam, Cat# ab216327, 1/100 dilution). It was then incubated with a goat anti-rabbit IgG H&L (Alexa Fluor^®^ 488) secondary antibody (Abcam, Cat# ab150077, 1/1000 dilution) and co-stained with Hoechst 33,258 (Sigma-Aldrich, Cat# B2883). Immunofluorescence signals were imaged using a confocal microscope (BX51TF, Olympus, Japan).

### Fluorescence *In Situ* Hybridization

As described previously (Manfredo Vieira et al. 2018), fluorescence *in situ* hybridization (FISH) was performed on spleen tissue sections using the pan-bacterial probe EUB338 (sequence: 5''-GCTGCCTCCCGTAGGAGT-3''; labeled with Cy5; Genscript). After DAPI staining, the sections were imaged using a confocal microscope (BX51TF, Olympus, Japan) and analyzed using ImageJ/FIJI software (National Institutes of Health, version 1.53c).

### Enzyme-linked immunosorbent assay (ELISA) Analysis

Peripheral blood was collected and serum was separated. The levels of anti-dsDNA antibodies, TNF-α, and IFN-γ in the serum were measured by ELISA. The anti-dsDNA ELISA kit was purchased from Shibayagi (Japan; Cat# 297-91801). The ELISA kits for TNF-α (Cat# AF02132-A) and IFN-γ (Cat# AF02182-A) were obtained from AiFang Biological (China). All assays were performed according to the manufacturer''s instructions.

### Quantitative real-time PCR (RT-qPCR)

The ileum was collected, and total RNA was extracted with RNA extraction reagent (Vazyme, Cat# R401) and subsequently reverse-transcribed into cDNA (Vazyme, Cat# R323-01). qPCR was conducted using SYBR Green reagents (Vazyme, Cat# Q711-02) on a QuantStudio 6 Flex system (Thermo), with β-actin as the internal control. Gene expression was calculated using the 2−ΔΔCt method. Primers were designed and synthesized by GenScript. The qPCR primer sequences are listed in Table S2.

###  16S rRNA Gene Amplicon Sequencing

Fecal samples underwent DNA extraction and 16S rRNA gene sequencing to facilitate the analysis of gut microbiota (data from one mouse in the MSC group were excluded from the 16S rRNA analysis due to insufficient fecal sample quantity). The sequencing library was constructed utilizing the TruSeq Nano DNA LT Library Preparation Kit (Illumina). Sequencing was executed on the NovaSeq 6000 platform (PE250, Illumina, USA). Raw sequences were processed into valid sequences and clustered into OTUs with > 97% similarity using UPARSE. OTU taxonomy was assigned via the SILVA database. Alpha diversity was analyzed with the Chao1 and Shannon indices, while beta diversity was evaluated using the weighted UniFrac distance. Permutational multivariate analysis of variance (PERMANOVA) was carried out to test whether the gut microbiome structure was significantly different. LEfSe was used for identifying microbial biomarkers.

### Targeted Metabolomics Profiling

Serum metabolite profiling was performed according to previously published methods (Xie et al. 2021). Briefly, serum samples (≥ 50 µL) were mixed with 200 µL ice-cold methanol containing isotope-labeled internal standards, vortexed for 30 s, incubated at − 20 °C for 20 min, and centrifuged (14,000 × *g*, 15 min, 4 °C). The supernatant underwent derivatization with 3-nitrophenylhydrazine (10 µL of 200 mM) and pyridine (40 µL) at 30 °C for 60 min, was quenched with formic acid, and was subsequently analyzed by UPLC-TQMS. Chromatographic separation was achieved using a C18 column (2.1 × 100 mm, 1.7 μm) with a gradient of mobile phase A (0.05% formic acid in water) and mobile phase B (acetonitrile) at 0.4 mL/min as follows: 5% B (initial) → 40% B (5 min) → 95% B (10–12 min) → 5% B (12.5–15 min). MS detection employed electrospray ionization in negative mode and multiple reaction monitoring (MRM) with an ion spray voltage of − 4,500 V and source temperature at 500 °C. Pooled quality control (QC) samples were injected every ten injections (with acceptance criteria of RSD < 15%), and absolute quantification of 324 metabolites was validated using 7-point calibration curves (0.1–1,000 µM; R² >0.990) with isotope internal standard correction. Data acquisition utilized MassLynx v4.1 (Waters, Milford, MA), and quantification was performed with TargetLynx v4.1. Metabolomic data analysis was conducted using MetaboAnalyst 6.0 (Pang et al. 2024).

### Statistical Analysis

Statistical analysis was performed using GraphPad Prism 9 (GraphPad, USA). Data from multiple *in vivo *experiments were analyzed using one-way analysis of variance (ANOVA) and the post hoc Tukey test. The amplicon sequencing data were analyzed using R software. Spearman correlation analysis was employed to examine the associations between cell subsets and metabolites or microbiota. To account for multiple comparisons, the Benjamini-Hochberg false discovery rate (FDR) correction was applied, with significance thresholds established at *p* < 0.05. Furthermore, the loading scores from partial least squares discriminant analysis (PLS-DA) were employed to identify metabolites with potential biomarker significance.

## Results

### Gut Microbiota Modification Via Co-housing Diminishes the Therapeutic Efficacy of MSC in Treating Lupus-Like Mice

**Fig. 1:**
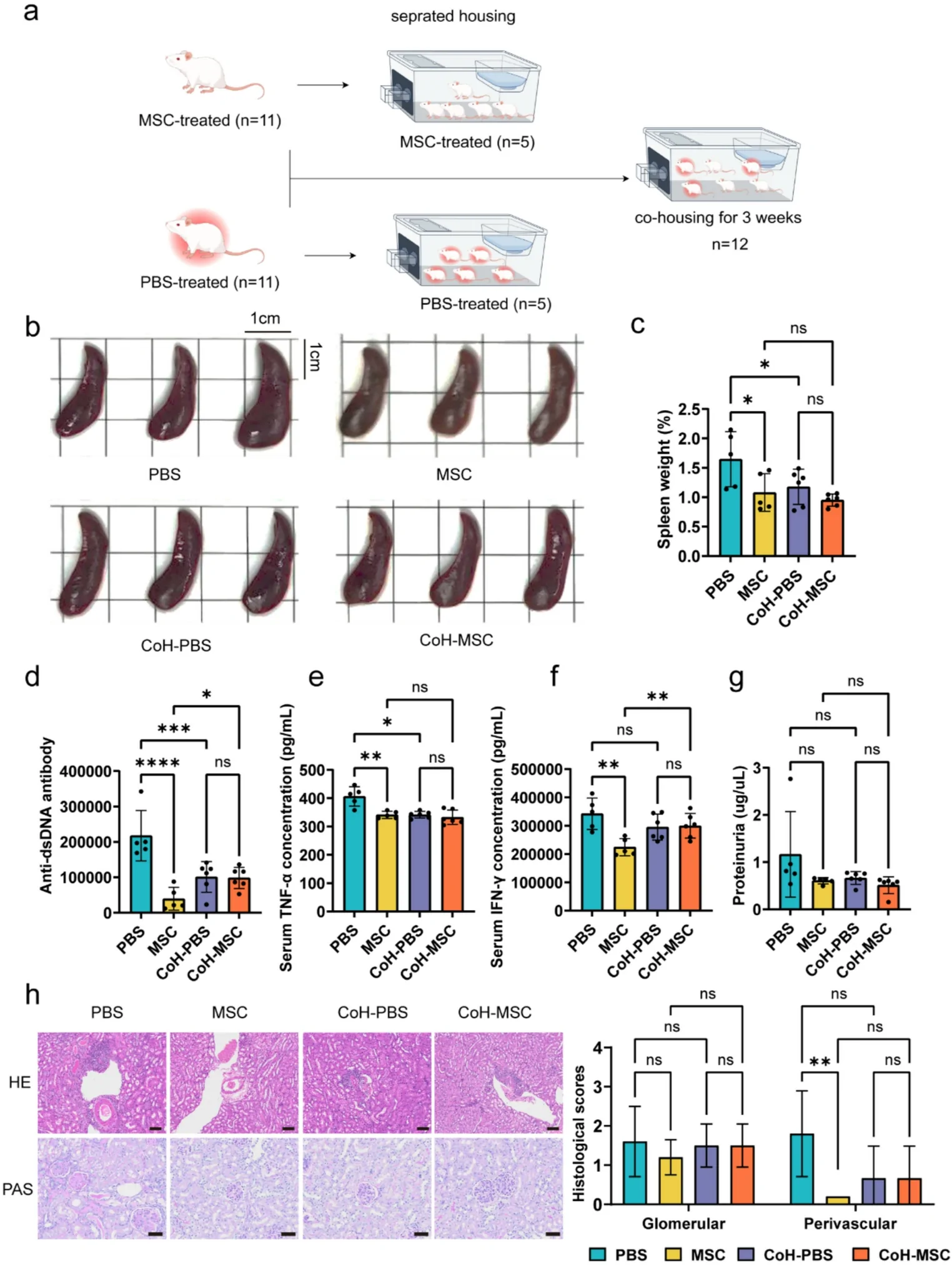
The impact of intestinal microbiota on the diminished therapeutic efficacy of MSC following co-housing. **a** Schematic of the experimental design for MSC treatment and co-housing. Following treatment and co-housing, the mice’s symptoms were assessed through various parameters: **b** spleen volume, **c** spleen-to-body weight index, **d** serum levels of anti-dsDNA antibodies measured by optical density at 450 nm, **e**-**f** serum concentrations of inflammatory cytokines TNF-α and IFN-γ, and **g** proteinuria levels. (*n* = 5–6). **h** Representative images of H&E (scale bar = 100 μm) and PAS (scale bar = 50 μm) -stained kidney sections, with three fields of view per section. Data were analyzed using ANOVA with Tukey''s post hoc test. *p < 0.05, **p < 0.01, ***p < 0.001, ****p < 0.0001. The results were presented as mean ± SD. CoH-PBS: co-housed PBS group, CoH-MSC: co-housed MSC group

First, we confirmed the therapeutic efficacy of MSC therapy in a lupus-like mouse model. Compared to the PBS control group, mice treated with MSC exhibited significant improvements in disease indicators. Specifically, MSC treatment led to a reduction in spleen size and a lower spleen-to-body weight index (Fig. 1b, c). Furthermore, MSC therapy markedly decreased the serum levels of anti-dsDNA autoantibodies and the pro-inflammatory cytokines TNF-α and IFN-γ (Fig. 1d-f), and showed a trend towards a reduction in proteinuria (Fig. 1g). Histopathological analysis of the kidneys confirmed these findings, revealing significantly ameliorated inflammatory cell infiltration around renal vessels in the MSC-treated group (Fig. 1h).

To explore the role of gut microbiota in the efficacy of MSC treatment, we conducted a co-housing experiment where MSC- and PBS-treated mice were housed together, allowing for microbial exchange (Fig. 1a). After three weeks, co-housing substantially altered the therapeutic outcomes. The protective effect of MSC on the spleen was compromised, as the difference in the spleen-to-body weight index between the co-housed groups was no longer significant (Fig. 1b, c). Co-housing also produced bidirectional effects on systemic and local inflammation. On one hand, it conferred therapeutic benefits to the PBS-treated mice; their serum levels of anti-dsDNA and TNF-α, as well as proteinuria, were significantly reduced compared to their non-co-housed counterparts (Fig. 1d, e, g). On the other hand, co-housing compromised the efficacy of MSC treatment. The co-housed MSC group exhibited increased levels of autoantibodies and IFN-γ compared to MSC-treated mice housed separately (Fig. 1d, f). These systemic changes were mirrored in the kidney pathology: while renal inflammation improved in the co-housed PBS group, the therapeutic improvements in the co-housed MSC group were partially reversed (Fig. 1h). These findings demonstrate that the therapeutic efficacy of MSC in this lupus-like model is critically dependent on the host’s gut microbiota.

### Co-housing Compromises the Immunomodulatory Effects of MSC on T and B Cell Subsets

**Fig. 2:**
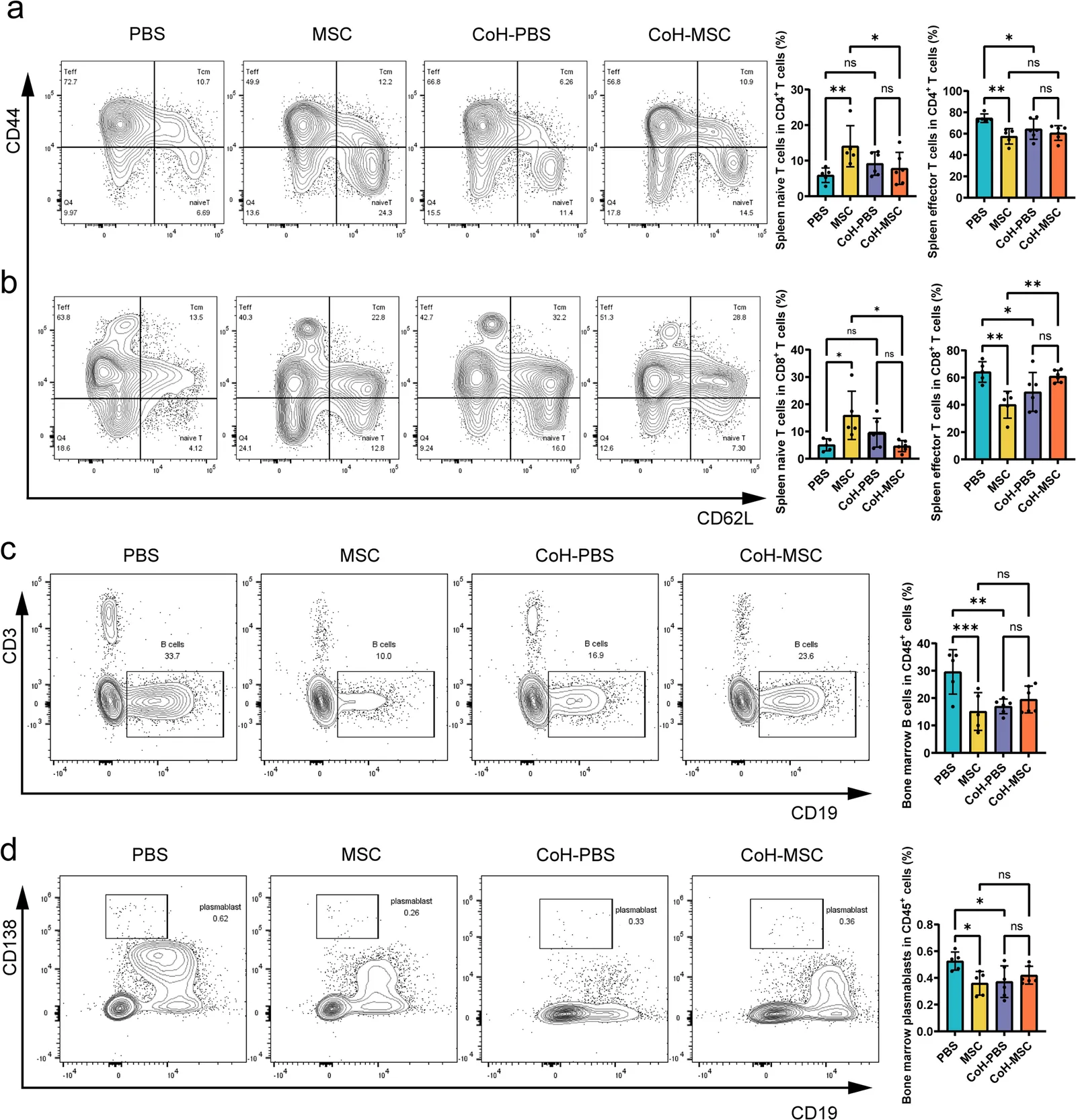
Co-housing reversed MSC-mediated immune cell rebalancing. Flow cytometric analysis of immune cell populations in the spleen and bone marrow. **a** The representative images and frequencies of naïve CD4^+^ T cells and CD4^+^ Teff cells in the spleen; **b** The representative images and frequencies of naïve CD8^+^ T cells and CD8^+^ Teff cells in the spleen. **c**-**d** Frequencies of total B cells and plasmablasts in the bone marrow. *n* = 5–6. Data were analyzed using one-way ANOVA with Tukey’s post hoc test. *p < 0.05, **p < 0.01, ***p < 0.001. The results were presented as mean ± SD. CoH-PBS: co-housed PBS group, CoH-MSC: co-housed MSC group

Given that T- and B- cell hyperactivation is a hallmark of lupus-like autoimmunity, we analyzed changes in splenic T lymphocytes and bone marrow B lymphocytes (Fig. S3). MSC treatment reshaped the lymphocyte proportions. In the spleen, this was characterized by an expansion of naïve CD4^+^ and CD8^+^ T cells and a concurrent reduction in their effector (Teff) counterparts compared to PBS-treated controls (Fig. 2a, b). Furthermore, this treatment also reduced the frequency of total B cells and plasmablasts in the bone marrow (Fig. 2c, d). Together, these data indicate that MSC effectively dampens T and B cell hyperactivation, thereby attenuating the generation of autoantibody-secreting cells.

However, these immunomodulatory effects were significantly altered by co-housing, which induced changes in the proportion of lymphocytes of both groups. In the MSC-treated group, the therapeutic benefits were attenuated; the previously observed reductions in CD4^+^ Teff, CD8^+^ Teff, and total B cells were partially reversed. Conversely, the co-housed PBS-treated mice acquired a partial therapeutic phenotype, exhibiting a significant decrease in the proportions of CD4^+^ Teff, CD8^+^ Teff, B cells, and plasmablasts. These results demonstrate that the gut microbiota is a critical mediator of MSC immunomodulation.

### Co-housing with Lupus-like Mice Diminishes the MSC-Induced Restoration of Intestinal Barrier Function and Increases Gut Permeability

**Fig. 3:**
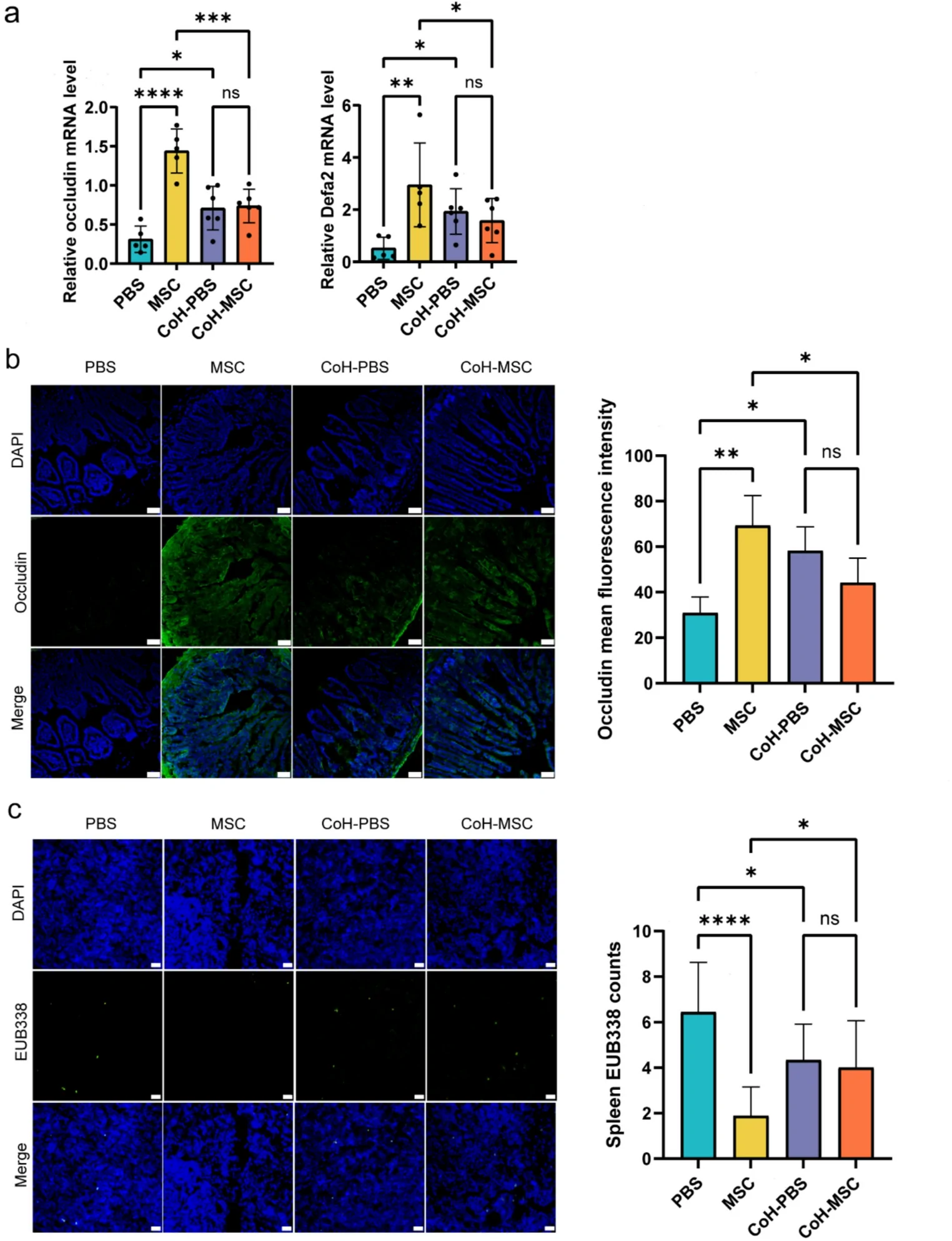
Co-housing reversed the intestinal barrier-restoring effects of MSC therapy in lupus-like mice. **a** Expression levels of the tight junction protein Occludin and the antimicrobial peptide Defa2 in the ileum (*n* = 5–6). **b** Representative immunofluorescence images showing Occludin (green) expression in ileum tissues. Quantification was performed by averaging five fields of view per sample (*n* = 3). Scale bar = 50 μm. **c** Representative FISH images using the pan-bacterial EUB338 probe (green) to visualize bacterial translocation into the spleen. Bacterial counts were quantified from three fields of view per sample (*n* = 3). Scale bar = 20 μm. In **b** and **c**, DAPI (blue) stains the nuclei. Data were analyzed using one-way ANOVA with Tukey''s post hoc test. *p < 0.05, **p < 0.01, ***p < 0.001, ****p < 0.0001. The data are presented as mean ± SD. CoH-PBS: co-housed PBS group, CoH-MSC: co-housed MSC group

Intestinal barrier dysfunction and the resultant "leaky gut" are recognized as pathogenic mechanisms in lupus. To investigate the gut barrier changes during MSC treatment, we measured the mRNA expression of *Occludin* and defensin alpha 2 (*Defa2*), two genes related to the intestinal barrier function. We found that MSC treatment led to an increase in the mRNA expression of *Occludin* and *Defa2* in the ileum. Moreover, after co-housing, the expression of *Occludin* and *Defa2* in the co-housed PBS group significantly increased compared to the control PBS group. Conversely, the expression of these genes in the co-housed MSC group significantly decreased compared to the MSC group. Moreover, there was no significant difference in gene expression between the two co-housed groups (Fig. 3a). We also used immunofluorescence to assess Occludin protein expression in the ileum, which corroborated the mRNA findings. MSC treatment increased Occludin protein expression in the ileum of lupus-like mice. Co-housing resulted in an increase in Occludin expression in the co-housed PBS group compared to the independently housed PBS group, while Occludin expression in the co-housed MSC group significantly decreased compared to the MSC group (Fig. 3b).

To evaluate the presence of leaky gut, we detected the translocation of intestinal bacteria using the EUB338 probe through FISH. The probe specifically hybridizes with a conserved region of the 16S rRNA gene common to bacterial species. The results demonstrated the presence of leaky gut in the disease model, and MSC therapy effectively reduced the EUB338 signal in the spleen, indicating enhanced intestinal barrier integrity in lupus-like mice. However, the reduced presence of EUB338 in the spleen of the co-housed PBS group compared with that in the independent PBS group also reflected the enhancement of the intestinal barrier after co-housing. In contrast, the EUB338 signal in the spleen of the co-housed MSC group significantly increased compared to the MSC group (Fig. 3c). These data demonstrate that the MSC-mediated enhancement of the intestinal barrier is contingent upon the host’s microbial state.

### Co-housing Mitigates MSC-induced Alterations in Gut Microbiota Composition

**Fig. 4:**
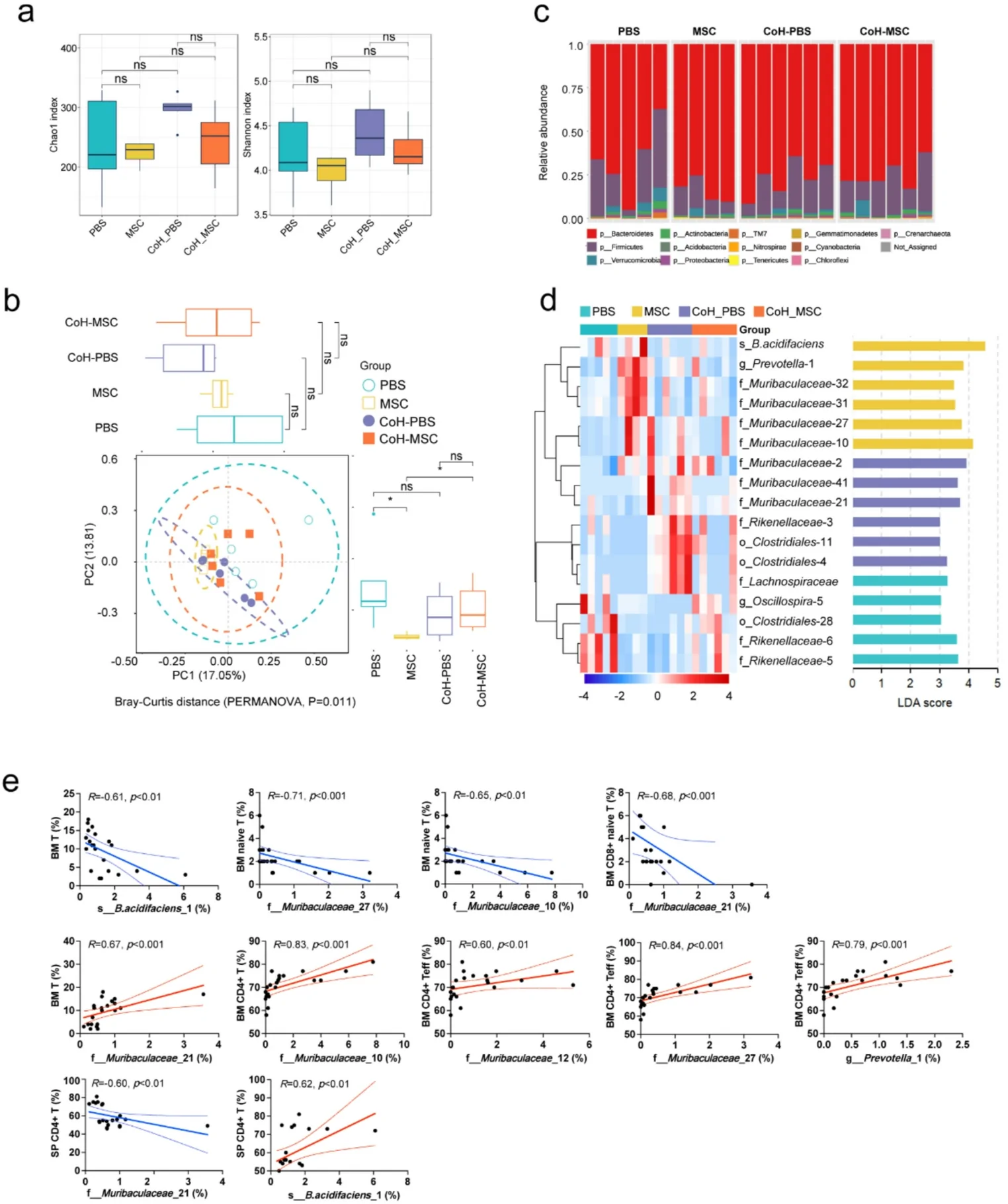
Co-housing changed MSC-mediated shifts in gut microbiota composition. Analysis of gut microbiota composition via 16S rRNA sequencing (*n* = 4–6 mice). **a** Alpha diversity metrics, including richness (Chao1 index) and diversity (Shannon index). **b** Principal coordinate analysis (PCoA) of beta diversity based on Bray-Curtis distances, showing distinct clustering of microbial communities. **c** Taxonomic composition showing the relative abundance of major bacterial phyla across groups. **d** Heatmap displaying differentially abundant taxa identified by linear discriminant analysis effect size (LEfSe) (|LDA score| ≥ 3, *p* < 0.05). **e** Results of Spearman rank correlation analysis results between bacterial genera and immune cell subsets in the BM and spleen. Spearman correlation coefficient |*R*| ≥ 0.6, *p* < 0.01. *n* = 4–6. CoH-PBS: co-housed PBS group, CoH-MSC: co-housed MSC group. p: phylum, o: order, f: family, g: genus, s: species

We conducted 16S rRNA sequencing to further investigate the influence of gut microbiota on MSC treatment efficacy. While MSC treatment did not significantly alter alpha diversity (Chao1 and Shannon indices), an increase was observed in both the co-housed PBS and co-housed MSC groups when compared to their respective pre-cohousing states (Fig. 4a). Beta diversity analysis with PCoA showed distinct clustering of bacterial communities between the PBS and MSC groups. Consistent with the phenotypic changes, the differences in the gut microbiota structure between the two groups appeared to be mitigated by the co-housing effect (Fig. 4b). At the phylum level, MSC treatment increased the relative abundance of *Bacteroidetes* while decreasing *Firmicutes*, *Actinobacteria*, and *Proteobacteria*. Notably, these MSC-induced compositional shifts were markedly diminished following co-housing (Fig. 4c).

Subsequently, we performed linear discriminant analysis effect size (LEfSe) to examine the differences in gut microbiota among the experimental groups at the OTU level. Compared with the PBS group, several taxa exhibited greater relative abundance in the MSC group, including one taxon from the *Prevotella* genus, 11 from the *Muribaculaceae* (previously known as *S24-7* family), and one from the *Bacteroidales* order (Fig. 4d; Table S3). Following co-housing, several taxa from the *Muribaculaceae*, which were initially more abundant in the MSC group, such as f_*Muribaculaceae-39*, f_*Muribaculaceae-18*, f_*Muribaculaceae-5*, f_*Muribaculaceae-27*, f_*Muribaculaceae-2*, f_*Muribaculaceae-12*, and f_*Muribaculaceae-10*, also demonstrated higher abundance in the co-housed PBS group (Fig. 4d; Table S4). The differences in gut microbiota between the two groups were reduced after co-housing, with similar relative abundances of the bacteria that were initially differentially abundant before co-housing (Table S6).

Finally, to link these microbial shifts to the observed immune changes, we performed Spearman correlation analysis between gut microbiota and lymphocyte subsets in both bone marrow (BM) and spleen (SP). We found significant correlations between numerous bacterial taxa and host immune cell proportions (Figs. S1a, S1b). In the BM, several taxa of the *Muribaculaceae* family demonstrated negative correlations with the proportion of naïve T cells (Spearman correlation coefficient *R* ≤ -0.6). Conversely, f_*Muribaculaceae-27*, along with a taxon from the *Prevotella* genus, was positively correlated with the proportion of CD4^+^ Teff cells (*R* ≥ 0.6) (Fig. 4e). In the spleen, the abundance of *f_Muribaculaceae-21* was strongly negatively correlated with CD4^+^ T cell proportions (*R* ≤ -0.6), whereas s_B.acidifaciens-1 showed a strong positive correlation (*R* ≥ 0.6) (Fig. 4e).

### Co-housing Alters MSC-induced Changes in Serum Metabolite Profiles in Lupus-like Mice

**Fig. 5:**
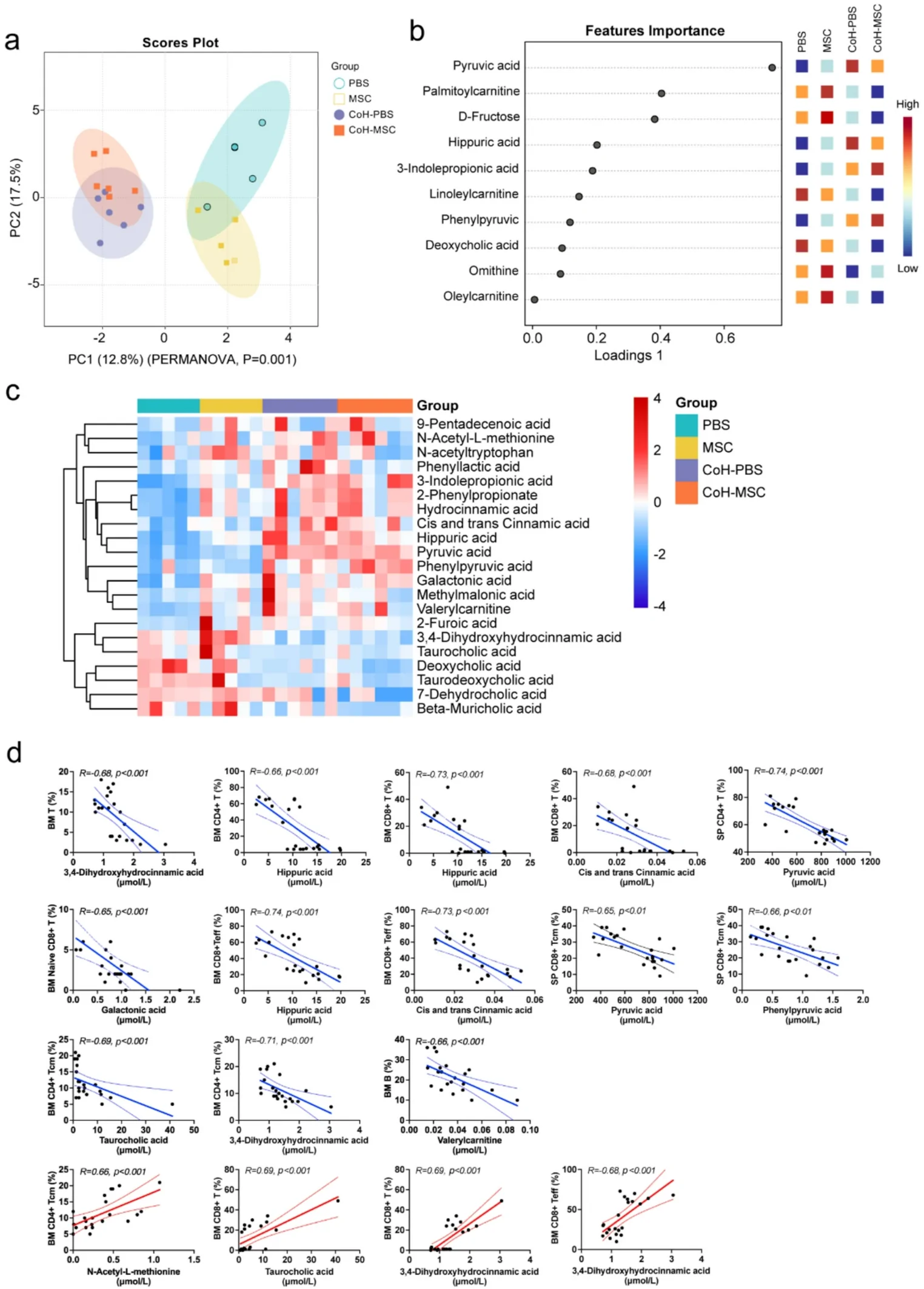
Co-housing treatment induced metabolic changes in the serum of lupus-like mice. **a** PLS-DA plots of serum metabolites among the groups (*p* < 0.05). **b** Loading plots of the 10 most significantly separated metabolites among the groups. **c** Heatmap displaying the differential metabolites between the groups. Differential metabolites were analyzed using the Wilcoxon rank-sum test: *p* < 0.05 and |log_2_FC| >1.0. **d** Results of Spearman rank correlation analysis results between metabolites and immune cell subsets in the BM and spleen. Spearman correlation coefficient |*R*| ≥ 0.65, *p* < 0.01. *n* = 5–6. CoH-PBS: co-housed PBS group, CoH-MSC: co-housed MSC group

Since the gut microbiota influences host physiology largely through its metabolic output, we next investigated the impact of MSC treatment and co-housing on serum metabolites. The PLS-DA analysis revealed a trend of separation in the metabolic profiles between the PBS group and the MSC group before co-housing. Conversely, following co-housing, the trend of metabolic profiling separation between the two groups was significantly attenuated (Fig. 5a). PA was a key contributor, with its levels significantly increasing in both groups after co-housing (Fig. 5b).

The metabolic profiling of lupus-like mice undergoing treatment with MSC therapy and subsequent co-housing demonstrated significant alterations in specific metabolites. Notably, MSC treatment resulted in marked increases in the concentrations of 2-furoic acid, galactonic acid, 9-pentadecenoic acid, hydrocinnamic acid, hippuric acid, 2-phenylpropionate, and N-acetyltryptophan in the lupus-like mice, thereby differentiating them from the control group (Table S7). Nevertheless, the marked disparities in metabolite levels observed between the treated and control groups were substantially reduced after co-housing (Table S8). Notably, 7-dehydrocholic acid was the only metabolite that demonstrated a significant difference between the co-housed PBS group and the co-housed MSC group. Following co-housing, the metabolites in the co-housed groups also underwent changes compared to those in the separately housed groups. Specifically, metabolites such as 2-furoic acid, galactonic acid, hydrocinnamic acid, 2-phenylpropionate, and hippuric acid, which were initially elevated in the MSC group prior to co-housing, demonstrated increased levels in the co-housed PBS group relative to the separately housed PBS group after co-housing (Table S9). At the same time, we observed that the overall levels of cinnamic acid (CA) , hippuric acid, and PA all increased after co-housing, while the level of DHCA actually decreased in the co-housed groups (Fig. 5c). Thus, the exchange of intestinal microbiota caused by co-housing led to metabolic changes in the lupus-like mice.

To uncover the functional relevance of these metabolic changes, we correlated the differential metabolites with immune cell subsets in the BM and SP. This analysis revealed extensive correlations (Figs. S2a, S2b), particularly between key metabolites andpathogenic T cell populations. For instance, in the BM, DHCA was positively correlated with CD8^+^ Teff cells, whereas CA and Hippuric acid were negatively correlated with this same cell subset. In the spleen, PA showed a strong negative correlation with both CD4^+^ T cells and CD8^+^ Tcm cells (Fig. 5d).

Collectively, these findings further suggest that changes in gut microbiota and metabolites are closely related to cellular-level alterations during MSC treatment of lupus-like autoimmunity.

### The Gut Microbiota-Metabolism Axis Modulates the Therapeutic Effects of MSC in Lupus-Like Autoimmunity

**Fig. 6:**
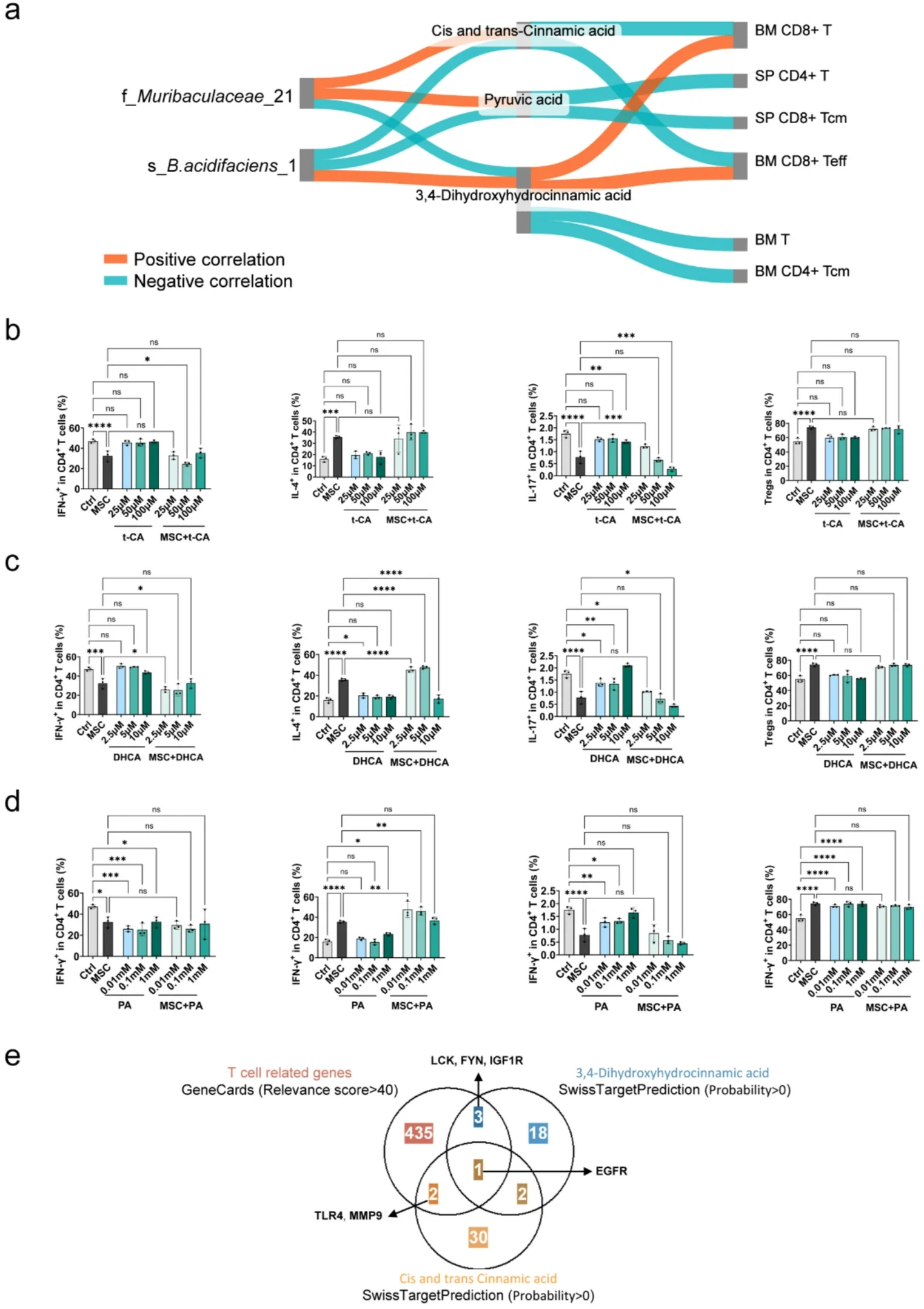
Serum metabolite profiles and gut microbiota signatures are associated with the immune-modulating effects of MSC therapy. **a** Sankey diagram visualizing the key associations between gut microbiota taxa, serum metabolites, and immune cell subsets identified *in vivo*. **b**-**d** In vitro analysis of naïve CD4^+^ T cell differentiation following co-culture with MSCs, key metabolites, or their combination. The specific metabolites tested were (**b)** t-CA, (**c)** DHCA, and (**d) **PA. (*n* = 3 per group). **e** Venn diagram of the intersection between metabolite targets and T cell-associated genes. Data were analyzed by one-way ANOVA with Tukey''s post hoc test. *p < 0.05, **p < 0.01, ***p < 0.001, ****p < 0.0001. p: phylum, o: order, f: family, g: genus, s: species. trans-cinnamic acid: t-CA, pyruvic acid: PA, 3,4-dihydroxyhydrocinnamic acid: DHCA

Our study investigated how the gut microbiota-metabolite axis influences the therapeutic effects of MSC in lupus-like autoimmunity. Using a Sankey diagram, we sought to identify the pathways through which gut microbiota modulate the development of T and B lymphocytes by altering host metabolic profiles. We observed a significant decrease in *Bacteroides acidifaciens* (*s_B.acidifaciens-1*) in the co-housed MSC group and a relative increase in a *Muribaculaceae* species (*f_Muribaculaceae-21*) in both co-housed groups (Tables S4, S5). These bacterial changes likely modulated serum levels of metabolites including CA, PA, and DHCA, significantly affecting T cell development in the bone marrow and spleen (Fig. 6a).

Furtherin vitro experiments confirmed the impact of these metabolites on MSC immunomodulatory function. Splenic T cells from R848-treated mice showed significantly increased activation compared to BALB/cJGpt mice, marked by increased proportions of CD4^+^ and CD8^+^ Teff cells (Figs. S3b-f). Co-culturing MSCs with these activated splenic T cells effectively suppressed T cell activation, inhibited naïve CD4^+^ T cell differentiation into Th1 and Th17 cells, and promoted the generation of immunosuppressive Tregs (Fig. 6b-d). This confirmed the in vitroimmunomodulatory capacity of MSCs. Using non-cytotoxic concentrations of t-CA, DHCA, and PA (Figs. S3a, S3c, S3e), we assessed their direct impact on T cell function. We found that t-CA significantly inhibited the activation of T cells into CD4^+^ Teff cells and reduced Th17 differentiation (Fig. 6b, S3b). Both DHCA and PA inhibited the activation of CD4^+^ and CD8^+^ T cells into effector T cells (Figs. S3d, S3e); DHCA suppressed Th17 while promoting Th2 generation (Fig. 6c), and PA suppressed Th1 and Th17 while significantly promoting Th2 and Treg differentiation (Fig. 6d). When these metabolites were present alongside MSCs, T cell activation was further reduced, the proportions of Teff cell (CD4^+^ and CD8^+^) were significantly decreased, and the regulation of Th1, Th2, and Th17 differentiation was enhanced. Specifically, MSCs with t-CA further lowered Teff cells (Fig. S3b) and inhibited Th17 at high concentrations (Fig. 6b); MSCs with DHCA further reduced CD4^+^ Teff cells (Fig. S3d), decreased Th1/Th17, and promoted Th2 at specific concentrations (Fig. 6c); MSCs with PA further reduced CD4^+^ Teff cells (Fig. S3f) and significantly promoted Th2 differentiation (Fig. 6d). This demonstrates that t-CA, DHCA, and PA synergistically enhance the immunosuppressive effects of MSC.

To further investigate the potential signaling pathways through which the gut microbiota-metabolite axis affects T cell development, we utilized SwissTargetPrediction to predict the gene targets of these metabolites. We found that CA may affect the functions of T cells via regulating *toll-like receptor 4* (*TLR4*), *matrix metallopeptidase 9* (*MMP9*), and *epidermal growth factor receptor* (*EGFR*) (Fig. 6e). Additionally, *lymphocyte cell-specific protein-tyrosine kinase* (*LCK*), *tyrosine-protein kinase Fyn* (*FYN*), *insulin-like growth factor 1 receptor* (*IGF1R*), and EGFR on T cells are potential targets of DHCA. The known functions of these genes in regulating the functions of T and B cells are summarized in Table 1.

**Table 1:** Regulation of T and B lymphocyte functions by the target genes of MSC treatment-modulated metabolites

Gene Symbol	Expression in immune subset	Influence on immune
**TLR4**	naïve CD4^+^ T	Proliferation Promotion (Reynolds et al. 2012)
	effector CD4^+^ T	Proliferation, Survival, Cytokine Production; Inhibition of Overactivation (Matharu et al. 2009; Rahman et al. 2009)
	Treg	Proliferation and Immunosuppression Promotion (Caramalho et al. 2003; Fukata et al. 2008)
	CD4^+^ T	Activation and Proliferation Mediation (Fukata et al. 2008; Reynolds et al. 2012); Th1/Th2 Imbalance Induction (Eisenbarth et al. 2002; Kang et al. 2023)
	γδ T	IL-17 and IFN-γ Production Promotion; Cell Proliferation (Eisenbarth et al. 2002; Kang et al. 2023)
	B cell	Activation, Plasma Cell Differentiation, and IL-10 Secretion (Eisenbarth et al. 2002; Kang et al. 2023)
**MMP9**	CD8^+^ T	Activation and Proliferation Promotion (Eisenbarth et al. 2002; Kang et al. 2023)
	CD4^+^ T	Activation and Proliferation Promotion (Benson et al. 2011)
	effector T	Inflammation Site Migration Regulation (Lavini-Ramos et al. 2017)
	Treg	Proliferation and Immunosuppressive Function Promotion (Lavini-Ramos et al. 2017)
**EGFR**	effector CD4^+^ T	Differentiation Promotion (Roy et al. 2021)
	Treg	Immunosuppressive Function Promotion (Kumagai et al. 2021)
**LCK**	iTreg	Promotion of differentiation upon LCK/Fyn inhibition (Qin et al. 2024)
	T cell	Phosphorylation-Dependent T-cell Activation (Zhou et al. 2018)
	B cell	IgM Secretion Promotion (Zhou et al. 2018)
**FYN**	Th17	Differentiation Promotion (Ueda et al. 2012)
	Treg	Th17/Treg Balance Regulation (Ueda et al. 2012)
	B cell	Autoantibody Production Promotion (Chaimowitz et al. 2013); Maturation Impact (Rodríguez-Alba et al. 2008)
	Plasma cell	Absence-Related Autoantibody Production Inhibition (Chaimowitz et al. 2013)
**IGF1R**	Th1	Immune Response Enhancement (Cecil et al. 2013)
	Treg	Proliferation Promotion (Kimura et al. 2018; Miyagawa et al. 2017)
	Plasma cell	Autoantibody Production Inhibition (Erlandsson et al. 2022)

In summary, our findings highlight the crucial role of the gut microbiota-metabolite axis in MSC therapy for SLE, where metabolites enhance MSC immunoregulation by modulating T cell activation and differentiation, potentially offering new targets to optimize the MSC treatment strategies.

## Discussion

In systemic lupus erythematosus (SLE), gut dysbiosis has been linked to increased intestinal permeability, allowing translocation of bacterial antigens into systemic circulation. This process triggers immune responses (Manfredo Vieira et al. 2018; Mu et al. 2017), including disrupted T cell differentiation and excessive B lymphocyte activation, ultimately driving autoantibody overproduction (Lian et al. 2024; Lopez et al. 2016; Shirakashi et al. 2022; Thim-Uam et al. 2020). The rebalancing of T and B cell populations and the enhancement of intestinal barrier function observed in MSC-treated mice indicate the gut microbiota''s role in mediating these immune-modulating effects. This study highlights the critical roles of the gut microbiota and their metabolites in modulating the therapeutic efficacy of MSC treatment for SLE. Using a lupus-like murine model, we demonstrate that gut microbiota modification significantly altered immune cell subsets, characterized by reduced naïve T cells and increased effector populations. These changes cumulatively compromised MSC efficacy, as evidenced by elevated anti-dsDNA antibodies, pro-inflammatory cytokines, and intestinal permeability. Further analysis identified *Bacteroides acidifaciens*, *Prevotella spp.*, and *Muribaculaceae spp.* as key mediators in the response to MSC therapy.

*B. acidifaciens*, a predominant commensal bacterium, promotes IgA production in the colon (Yanagibashi et al. 2013), modulates host insulin resistance and energy metabolism via autophagy regulation (Yang et al. 2017), and maintains barrier homeostasis by stimulating mucus secretion and upregulating tight junction proteins (Fan et al. 2025). Notably, in autoimmune hepatitis models, *B. acidifaciens* uniquely mitigates liver injury by downregulating hepatocyte CD95 (Fas) expression, suppressing T cell-mediated apoptosis—an effect not observed in other *Bacteroides* species (e.g., *B. thetaiotaomicron*), indicating strain-specific protection (Wang et al. 2022). The *Prevotella* genus (of Gram-negative anaerobes) exhibits altered abundance in immune disorders; for instance, *Prevotella* levels inversely correlate with multiple sclerosis severity (Mangalam and Murray 2019). Oral administration of *P. histicola* suppresses disease via induction of immunoregulatory cells and inhibition of pro-inflammatory responses (Mangalam et al. 2017). *Muribaculaceae* depletion is linked to inflammatory bowel disease, obesity, and type 2 diabetes (Zhu et al. 2024). *Muribaculaceae* loss under high-fat diets is associated with impaired barrier function and dysbiosis (Tomas et al. 2016). Thus, MSC-induced enrichment of beneficial bacteria likely reduces antigenic load and pro-inflammatory cytokines (e.g., IFN-γ), restoring barrier integrity in SLE.

MSC efficacy was diminished under co-housing conditions due to coprophagy-driven microbiota transfer (Bo et al. 2020), which resulted in convergence of the microbial profiles between MSC-treated and control mice. This reversed key therapeutic benefits, including lymphocyte re-balancing and barrier restoration. Serum metabolite alterations correlated closely with these microbiota shifts. *Muribaculaceae* metabolizes endogenous (mucin glycans) and exogenous (dietary fiber) polysaccharides to produce short-chain fatty acids ). Utilizing sugars released following polysaccharide hydrolysis as precursor, this process generates pyruvate through polysaccharide hydrolysis and glycolysis to maintain metabolic flux, thereby increasing PA levels. By shaping the gut environment and cross-feeding with microbes like *Bifidobacterium* and *Lactobacillus* (which metabolize compounds like CA and its derivatives) ), *Muribaculaceae* may indirectly influence CA and DHCA breakdown (Zhu et al. 2024).

Among perturbed metabolites, several immunomodulatory compounds affected MSC therapy outcomes. CA derivatives (e.g., cinnamaldehyde) exhibit immunosuppressive effects by inhibiting immune cell proliferation and promoting apoptosis, preferentially impacting T cells over B cells (Roth-Walter et al. 2014). Our in vitro validation confirmed that CA reduces T cell activation and promotes balanced Th1/Th2 and Th17/Treg profiles. Similarly, PA alters lymphocyte metabolism, affecting activation and proliferation via glycolysis (Israelsen and Vander Heiden 2015; Liu et al. 2014) and intracellular signaling (Assmann and Finlay 2016). We confirmed that PA suppresses T cell activation. DHCA also demonstrated efficacy in improving T cell balance in vitro, potentially by regulating the Th1/Th2 and Treg/Th17 balance (Zhou et al. 2022). Our in vitro experiments showed that DHCA effectively reduced T cell activation and improved T cell balance. Crucially, co-culture with MSCs revealed a synergistic effect of CA, DHCA, and PA on T cell regulation, suggesting these metabolites may enhance MSC immunomodulation. Diminished levels of hydrocinnamic acid (a *Prevotella*-derived metabolite) attenuate aryl hydrocarbon receptor activation, downregulating tight junction proteins (ZO-1, E-cadherin, connexin 43) and compromising barrier function (Hu et al. 2023). These functional insights, combined with the influence of host metabolic status on MSC efficacy (Sharon et al. 2014), highlight a mechanistic interplay between microbiota-derived metabolites and immune regulation—specifically the direct modulation of T cells by these metabolites and their potential to optimize MSC therapy (Simms-Waldrip and Koh 2018).

We further identified immune cell targets of MSC-response metabolites. *TLR4* upregulation may exacerbate SLE inflammation (Lartigue et al. 2009; Richez et al. 2011), making TLR4 antagonists or monoclonal antibodies promising therapeutics (Kalliolias et al. 2024). *MMP9* deficiency in SLE models impairs clearance of autoantigens and immune complexes, suggesting its protective role (Ugarte-Berzal et al. 2019). Additionally, blocking *EGFR* signaling amplifies T cell responses and lupus nephritis severity, implicating the *Amphiregulin* (*AREG*)/*EGFR* axis in disease regulation (Melderis et al. 2022).

### Conclusions and Limitations

Our study establishes the gut microbiota as a key modulator of MSC efficacy in a lupus-like model. While our study provides some insights into the interplay between gut microbiota and MSC therapy, several limitations should be acknowledged. First, the exact mechanisms through which specific microbial species and their metabolites influence immune modulation remain unclear, and future work should delineate these critical microbial players and metabolic pathways. Second, while co-housing demonstrated microbiota transfer effects, controlled methods (e.g., fecal microbiota transplantation) would better isolate the specific contributions. Furthermore, to enhance the generalizability of our findings, validation across different established lupus-prone mouse models is warranted to confirm the consistency of the gut microbiota-MSC interaction. Additionally, findings require validation in human cohorts, given SLE heterogeneity, genetic backgrounds, disease stages, and environmental factors affecting microbiota-MSC interactions. Despite these limitations, our results suggest gut microbiome modulation as a promising adjunctive strategy to enhance immunomodulatory therapies for autoimmune diseases.

## Supplementary Information

Below is the link to the electronic supplementary material.

Supplementary Material 1

Supplementary Material 2

## Data Availability

The 16S rRNA raw data generated in this study have been deposited in the China National Center for Bioinformation, with the GSA number CRA028565. The full list of pathogens excluded in the SPF animal facility supporting this study is available from the corresponding author upon reasonable request.

## Abbreviations

IBD: Inflammatory bowel disease
FMT: Fecal microbiota transplantation
ICIs: Immune checkpoint inhibitors
UC-MSC: Umbilical cord-derived mesenchymal stromal cell
SPF: Specific pathogen-free
ECTV: Ectromelia virus
MHV: Mouse hepatitis virus
SV: Sendai virus
PVM: Pneumonia virus of mice
MVM: Minute virus of mice
MNV: Murine norovirus
LCMV: Lymphocytic choriomeningitis virus
R848: Resiquimod
H&E: Hematoxylin and eosin
PAS: Periodic acid-Schiff
GBM: Glomerular basement membrane
CA: Cinnamic acid
t-CA: Trans-Cinnamic acid
PA: Pyruvic acid
DHCA: 3,4-Dihydroxyhydrocinnamic acid
FISH: Fluorescence *in situ* hybridization
ELISA: Enzyme-linked immunosorbent assay
RT-qPCR: Reverse transcription quantitative real-time PCR
PERMANOVA: Permutational multivariate analysis of variance
ANOVA: Analysis of variance
FDR: False discovery rate
PLS-DA: Partial least squares discriminant analysis
Teff: Effector T cell
Tcm: Central memory T cell
Defa2: Defensin alpha 2
LEfSe: Linear discriminant analysis effect size
BM: Bone marrow
SP: Spleen
SLE: Systemic lupus erythematosus
TLR4: Toll-like receptor 4
MMP9: Matrix metallopeptidase 9
EGFR: Epidermal growth factor receptor
LCK: Lymphocyte cell-specific protein-tyrosine kinase
IGF1R: Insulin-like growth factor 1 receptor
FYN: Tyrosine-protein kinase Fyn
