# Supplementary material for: The Role of Intestinal Microbiota in Modulating Mesenchymal Stromal Cell Therapy in a Murine Model of Lupus-like Autoimmunity
Source: Phenomics. 2026 Sep 7;6(5):635–51. doi: 10.1007/s43657-025-00303-z (PMC13743704; doi:10.1007/s43657-025-00303-z)
Supplement: Supplementary file 1 [file 43657_2025_303_MOESM1_ESM.docx]

**Supplementary figure 1. Correlation analysis between microbial taxa and immune cell subsets.** Heatmaps display the Spearman rank correlation between the relative abundances of microbial taxa and the frequencies of immune cell subsets in the (a) BM and (b) spleen. |*R*| ≥ 0.5 and *p* < 0.05. **p* < 0.05; ***p* < 0.01; ****p* < 0.001. Taxonomic levels are abbreviated as follows: p, phylum; o, order; f, family; g, genus; s, species.

**Supplementary Figure 2. Correlation analysis between metabolites and immune cell subsets.** Heatmaps display the Spearman rank correlation between metabolite levels and the frequencies of immune cell subsets in the (a) BM and (b) spleen. |*R*| ≥ 0.5 and *p* < 0.05. **p* < 0.05; ***p* < 0.01; ****p* < 0.001.

**Supplementary figure 3. Functional impact of metabolites doses on T cell viability and activation under MSC co-treatment.** (a) The influence of different concentrations of t-CA on the viability of T cells. (b) The effects of varying concentrations of t-CA and MSCs on T cell activation. (c) The influence of different concentrations of DHCA on the viability of T cells. (d) The effects of varying concentrations of DHCA and MSCs on T cell activation. (e) The influence of different concentrations of PA on the viability of T cells. (f) The effects of varying concentrations of PA and MSCs on T cell activation. By one-way ANOVA with Tukey’s post-hoc test. **p* < 0.05, ***p* < 0.01, ****p* < 0.001, *****p* < 0.0001. Ctrl group: T cells from spleen of BALB/cJGpt mice; other groups: T cells from spleen of R848-induced mice.

**Supplementary figure 4. Flow cytometry gating strategy**. (a) Bone marrow T/B cell gating strategy. (b) Spleen T/B cell gating strategy. (c) Th1 cell gating strategy. (d) Th2 cell gating strategy. (e) Th17 cell gating strategy. (f) Treg cell gating strategy.
